# Neuroprotection of cannabidiol in epileptic rats: Gut microbiome and metabolome sequencing

**DOI:** 10.3389/fnut.2022.1028459

**Published:** 2022-11-16

**Authors:** Xiaoxiang Gong, Lingjuan Liu, Xingfang Li, Jie Xiong, Jie Xu, Dingan Mao, Liqun Liu

**Affiliations:** ^1^Department of Pediatrics, The Second Xiangya Hospital, Central South University, Changsha, Hunan, China; ^2^Children’s Brain Development and Brain Injury Research Office, The Second Xiangya Hospital, Central South University, Changsha, Hunan, China

**Keywords:** Epilepsy, cannabidiol, gut microbiota, metabolism, neuroprotection

## Abstract

**Aims:**

Epilepsy is a neurological disease occurring worldwide. Alterations in the gut microbial composition may be involved in the development of Epilepsy. The study aimed to investigate the effects of cannabidiol (CBD) on gut microbiota and the metabolic profile of epileptic rats.

**Materials and methods and results:**

A temporal lobe epilepsy rat model was established using Li-pilocarpine. CBD increased the incubation period and reduced the epileptic state in rats. Compared to epileptic rats, the M1/M2 ratio of microglia in the CBD group was significantly decreased. The expression of IL-1β, IL-6, and TNF-α in the CBD group decreased, while IL-10, IL-4, and TGF-β1 increased. 16S rDNA sequencing revealed that the ANOSIM index differed significantly between the groups. At the genus level, *Helicobacter*, *Prevotellaceae_UCG-001*, and *Ruminococcaceae_UCG-005* were significantly reduced in the model group. CBD intervention attenuated the intervention effects of Li-pilocarpine. *Roseburia*, *Eubacterium_xylanophilum_group*, and *Ruminococcus_2* were strongly positively correlated with proinflammatory cytokine levels. CBD reversed dysregulated metabolites, including glycerophosphocholine and 4-ethylbenzoic acid.

**Conclusion:**

CBD could alleviate the dysbiosis of gut microbiota and metabolic disorders of epileptic rats. CBD attenuated Epilepsy in rats might be related to gut microbial abundance and metabolite levels.

**Significance and impact of study:**

The study may provide a reliable scientific clue to explore the regulatory pathway of CBD in alleviating Epilepsy.

## Introduction

Epilepsy is a chronic nervous system disease that affects at least 70 million people worldwide (1). Temporal lobe epilepsy (TLE) is the most common type of focal Epilepsy worldwide. In the TLE model, inflammation is one of the most upregulated biological processes in Epilepsy (2). Inflammatory factors released by immune cells directly or indirectly affect neuronal excitability, thereby regulating the threshold of epileptic seizures (3). Neuroinflammation is considered the main pathological finding of Epilepsy. Microglia are highly adaptable glial cells in the central nervous system (CNS) and play an important role in maintaining CNS homeostasis (4). Epilepsy triggers the rapid activation of nearby microglia. The excess inflammatory mediators produced by activated microglia may promote the inflammatory immune cascade (5, 6). New evidence has shown that neuroinflammation can affect hyperexcitability and promote Epilepsy (7). Meanwhile, M2 microglia polarization could alleviate seizures *via* suppressing neuronal apoptosis and the hyperactivation of M1 microglia (8–10). Therefore, neuroinflammation and M2 microglia polarization may be an important pathophysiological mechanism in the development of TLE.

Cannabidiol (CBD) is the main active ingredient in cannabis. Unlike tetrahydrocannabinol (THC), CBD does not exhibit excitation-inducing properties. CBD has antioxidant and anti-inflammatory activities and could play a neuroprotective role by modulating the biological targets of the brain in neurodegenerative diseases (11). Clinical studies have reported that CBD has a good therapeutic effect in relieving pain and treating Epilepsy (12). Some enzymes, ion channels, receptors, and transporters, including G protein-coupled receptor (GPR), are molecular targets for CBD therapy (13). However, how CBD regulates neuroinflammation in the brain and alleviates the development of Epilepsy and its internal regulatory pathways remain unclear.

Gut microbiota can regulate gut permeability, alter local or peripheral immune responses, and produce essential metabolites and neurotransmitters (14). Moreover, gut microbiota can achieve “gut-brain” communication through endocrine, immune and metabolic pathways (15). There is evidence that the composition of gut microbiota changes during Epilepsy (16), and antiepileptic drugs can affect gut microbiota (17). Recent studies have shown that the anti-inflammatory properties of CBD may be involved in resisting gut inflammation, leakage of the gut vascular barrier caused by dysregulation of the gut microbiome, and subsequent neuroinflammation (18). However, the effects of CBD on gut microbiota during Epilepsy treatment have been rarely reported.

We speculated that CBD might inhibit the overexpression of inflammatory factors in Epilepsy by reducing the activation of microglia. In addition, we will explore whether CBD mediates changes in the composition and function of gut microbiota in epileptic rats. We hope that this study provides preliminary reliable scientific evidence in support of further investigations to explore the regulatory pathway of CBD in alleviating Epilepsy.

## Materials and methods

### Animal

Fifty Sprague-Dawley male rats were purchased from Hunan SJA Laboratory Animal Co., Ltd., Rats were adaptively fed for 5 days (d) under specific pathogen-free conditions with a controlled 12 h light/dark cycle, temperature (20–25°C), humidity (50–60%), and free access to water and diet. The rats were randomly divided into the following five groups: a control group (control group) (*n* = 10), an epilepsy model group (*n* = 10), a low-CBD epilepsy model group (Model + 20 mg/kg CBD group) (*n* = 10), a high-CBD epilepsy model group (Model + 100 mg/kg CBD group) (*n* = 10), and a Carbamazepine (CBZ) epilepsy model group (Model + 75 mg/kg CBZ group) (*n* = 10). CBD and CBZ were purchased from Sigma-Aldrich, St. Louis, MO, USA. We chose a dose of 20 mg/kg based on a previous study demonstrating that this was within the range of anti-inflammatory therapy in rodents and humans (19). A second study also demonstrated that 100 mg/kg of CBD exerted a significant antiepileptic effect in rodent models of Epilepsy (20). CBZ could relieve seizures with CBZ 75 mg/kg injected (21).

Model treatment: The rats were intraperitoneally injected with 127 mg/kg lithium chloride the day before modeling (day 6). After 18–24 h, freshly prepared pilocarpine (1538902, Sigma-Aldrich, St. Louis, MO, USA) was intraperitoneally injected at a ratio of 25 mg/kg. The control group was injected with the same volume of solvent. The rats were subcutaneously injected with 0.1 mg atropine sulfate monohydrate 30 min before the injection of pilocarpine. Seizure severity was graded according to the Racine Scale (22). Rats with recurrent epileptic lasting 30 min were considered to have status epilepticus (SE). Rats with SE were intraperitoneally injected with 10 mg/kg of Diazepam to terminate the attack (23, 24).

The low-CBD group was administered 20 mg/kg CBD by gavage half an hour before modeling. The high-CBD group was administered 100 mg/kg CBD. The CBZ group was injected at 75 mg/kg CBZ half an hour before modeling. The control and model groups were administered the same amount of carrier solution (2% Tween 80 + 98% saline). The rats were treated continuously for 7 days. Under the experimental animal ethics protocol, all rats were intraperitoneally injected with chloral hydrate for euthanasia. Brain tissue and feces were collected. The experiments on rats in this study were approved by the Animal Ethical and Welfare Committee, The Second Xiangya Hospital, Central South University (No. 2021523).

### Hematoxylin and eosin staining

Rat brain tissue was collected and fixed with 4% paraformaldehyde for 24 h. After treatment with xylene and graded alcohol, samples were stained with hematoxylin for 10 min. Next, the samples were incubated with eosin staining solution for 5 min. Samples were dehydrated through graded alcohol. Neutral resin was used for sealing. A light microscope was used for observation, and images were obtained.

### Immunofluorescence

Rat brains were sectioned and dewaxed. The slices were then dipped in ethylene diamine tetraacetic acid (EDTA) buffer (pH 9.0) for thermal repair. 3% H_2_O_2_ was used to inactivate endogenous enzymes, and phosphate-buffered saline (PBS) was used for flushing. Primary antibody microglia/macrophage-specific protein IBA1 (MA5-27726, Invitrogen, Carlsbad, CA, USA) was added and diluted at a ratio of 1:100, incubated overnight at 4°C, and washed with PBS. An appropriate amount of anti-mouse-IgG-labeled fluorescent antibody was added, incubated at 37°C for 90 min, and rinsed with PBS. The DAPI working solution was stained at 37°C for 10 min. Buffered glycerin was used to seal the tablets, which were then observed under a fluorescence microscope.

### Flow cytometry

Brain tissue was trypsinized to obtain cells. Samples, about 1 × 10^6^ cells per well, were placed in a 1.5 ml centrifuge tube, and the cells were resuspended in 200 μl PBS. The 5 μl of CD45 (12-0461-82, eBioscience, USA) and CD86 (374215, BioLegend, San Diego, CA, USA), or CD45 and CD163 (326511, BioLegend, San Diego, CA, USA) were added to the sample. The mixture was then incubated in the dark for 30 min. The cells were washed twice with 1 ml of PBS. Then cells were resuspended in 200 μl PBS and filtered through a nylon mesh. Flow cytometry was used for detection.

### Quantitative real-time PCR

Total RNA was extracted from the temporal lobe cortex using the TRIzol™ method. The TRIzol Reagent was purchased from ThermoFisher scientific. RNA concentration was measured using an ultraviolet spectrophotometer. According to the instructions of the Hifiscript cDNA Synthesis Kit (CW2569M, CWBIO, Beijing, China), a 20 μl reaction system was used for reverse transcription. PCR amplification was performed according to the UltraSYBR Mixture (CW2601, CWBIO, Beijing, China) manual, and the reaction volume was 30 μl. The SYBR method was used for qPCR detection, and the primers were synthesized by Sangon Biotech. Specific primer sequences are as follows: Rat-β-actin, F-ACATCCGTAAAGACCTCTATGCC, R-TACTCCTGCTTGCTGATCCAC, product length 223bp; Rat-IL-1β, F-CAGCAGCATCTCGACAAGAG, R-AAAGAAGGTGCTTGGGTCCT, product length 123bp; Rat-IL-6, F-TCACTATGAGGTCTACTCGG, R-CATATTGCCAGTTCTTCGTA, product length 141bp; Rat-TNF-α, F-CCCCTCTATTTATAATTGCACCT, R-CTGGTAGTTTAGCTCCGTTT, product length 167bp; Rat-IL-10, F-AATAAGCTCCAAGACAAAGGT, R-TCACGTAGGCTTCTATGCAG, product length 79bp; Rat-IL-4, F-ATGCACCGAGATGTTTGTACC, R-GACCGCTGACACCTCTACAGA, product length 185bp; Rat-TGF-β1, F-ACTACGCCAAAGAAGTCACC, R-CACTGCTTCCCGAATGTCT, product length 125 bp. Data were normalized relative to the control group, and β-actin was used as an internal reference. The 2^–ΔΔ^ ^Ct^ reflects the ratio of each sample’s target gene expression level relative to that of the control group.

### Western blotting

The detection of temporal lobe cortex proteins was undertaken as previously described (25). Radio Immunoprecipitation Assay (RIPA) lysate (P0013B, Beyotime, Shanghai, China) was used to extract total protein from temporal lobe cortex tissue. After lysed for 10 min on ice, the tissue homogenate was centrifuged at 12,000 rpm, 4°C for 10 min. The supernatant was boiled in water for 5 min to denature the protein. Denatured proteins were separated on 10% SDS-polyacrylamide gels and transferred to nitrocellulose (NC) membranes. After blocking with non-fat milk, the membranes were incubated with the primary antibody overnight at 4°C. The secondary antibody was incubated for 90 min. Images were obtained using a chemiluminescence imaging system. The antibodies used in this study were as follows: IL-1β (16806-1-AP,1:1000, Proteintech, Chicago, IL, USA), IL-6 (M620,1:1000, Invitrogen, Carlsbad, CA, USA), TNF-α (17590-1-AP,1:500, Proteintech, Chicago, IL, USA), IL-10 (20850-1-AP,1:1000, Proteintech, Chicago, IL, USA), IL-4 (66142-1-Ig,1:1000, Proteintech, Chicago, IL, USA), TGF-β1 (21898-1-AP,1:1000, Proteintech, Chicago, IL, USA), β-actin (60008-1-Ig,1:5000, Proteintech, Chicago, IL, USA), HRP goat anti-mouse IgG (SA00001-1,1:5000, Proteintech, Chicago, IL, USA), and HRP goat anti-rabbit IgG (SA00001-2,1:6000, Proteintech, Chicago, IL, USA).

### 16S rDNA sequencing

16S rDNA sequencing was performed on fecal samples from 20 rats, with five rats in each group. A NovaSeq PE250 instrument (Illumina, San Diego, CA, USA) was used for 16S amplicon sequencing to obtain raw data. DADA2 performed low-quality filtering operations, such as adapters, primer removal, de-noising, merging paired-end sequences, and removing chimeras to obtain valid data. The quality control standard was set as truncQ = 2, F maxEE = 5, R maxEE = 2. The sequencing depth was 50,000 reads, and one read was 250 bp. In order to obtain species classification information, according to the silva-132-99 database, the amplification primer Bacterial V3/V4 (341F + 805R) in the V3–V4 region was selected, and the primer sequence was: CCTACGGGNGGCWGCAGGACTACHVGGGTATCTAATCC. Clusters that reached 99% features of the reference genome Silva132 were grouped into the same taxon. QIIME 2 (Qiime2-2020.2) was used to calculate the α-diversity indices of the samples. The R Phyloseq/Vegan package, Deseq2 package, and Jvenn^1^ online software were used for visualization.

### Untargeted metabolomics analysis

For investigating the effects of CBD on fecal metabolite levels in epileptic rats, 20 fecal samples were analyzed using untargeted metabolomics analysis. The rats were divided into control, model, low-CBD, and high-CBD model groups, with five rats in each group. Samples were quickly pounded using a plastic hammer. A fecal sample (approximately 50 mg) was collected from each rat and weighed. Nine volumes of the extract containing an internal standard of ^13^C stable isotope were added to each sample. The sample was stored on ice for 15 min to lyse cells fully. The mixture was centrifuged at 16,000 *g* at 4°C for 10 min. The supernatant was transferred to a new centrifuge tube, and nitrogen was used to dry the samples. After rescaling, the supernatant of the extract was used for the injection analysis. A TripleTOF 5600 + MS system (AB Sciex, Framingham, MA, USA) and an Acquity UPLC HSS T3 column (Waters) were used for LC-MS analysis.

### Data analyses

Statistical software SPSS 23.0 and GraphPad Prism 8.0.1 were used to analyze the data in this study. Data are presented as the mean ± standard deviation. A Kruskal–Wallis test, one-way ANOVA, and two-way ANOVA were used to compare groups. Metabolomics data analysis was completed online using MetaboAnalyst5.0.^2^ Spearman’s rank test was used to analyze the correlation between different indicators, and statistical significance was set at *P* < 0.05.

## Results

### Cannabidiol alleviates epilepsy in rats

**FIGURE 1:**
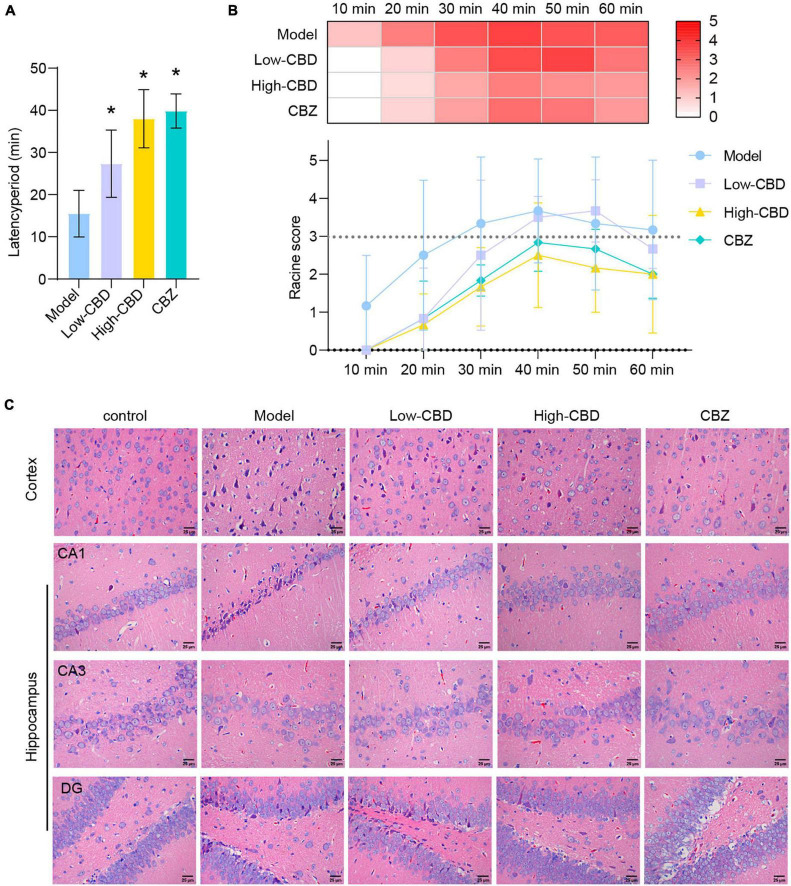
Cannabidiol (CBD) alleviates Epilepsy in rats. **(A)** The latency period of epileptic rat. **(B)** The Racine score within 1 h of modeling. **(C)** H&E staining results of the temporal lobe cortex and hippocampus. *Represents significant comparison with the Model group, *P* < 0.05. The darker the red, the higher the Racine score.

After modeling, we found that the rats in the model group had the earliest seizures, at 15.5 min on average, followed by the low-CBD group at 27.3 min on average, and the slowest in the high-CBD group at 38.0 min on average (Figure 1A). Statistics of epileptic rats’ seizures within 80 min demonstrated. The model group had grade 4–5 seizures within 30 min, then continued to have grade 3–4 seizures. The rats required anesthesia to stop the seizures forcibly. The low-CBD group had grades 3–4 seizures within 40 min, occasional grades 2–3 seizures within 50–70 min, and occasional grades 1–2 seizures within 70–80 min, and there was no need to terminate the seizures forcibly. The high-CBD group had grades 3–4 within 60 min, and 1–2 seizures occasionally occurred within 60–80 min, without the need for forced termination of seizures (Figure 1B). Compared with the CBZ group, the high-CBD group had no significant difference in latency and seizure statistics within 80 min. H&E staining was performed to analyze the pathological morphology of temporal lobe cortex and hippocampus (Figure 1C). Compared with the control group, the model group showed significant histopathological changes, including neuronal atrophy and pyknosis. The morphological damage of brain tissue was improved in the CBD group and the CBZ group. From this, we believed that CBD could relieve Epilepsy in rats.

### Cannabidiol promotes M2-type polarization in epileptic rats

**FIGURE 2:**
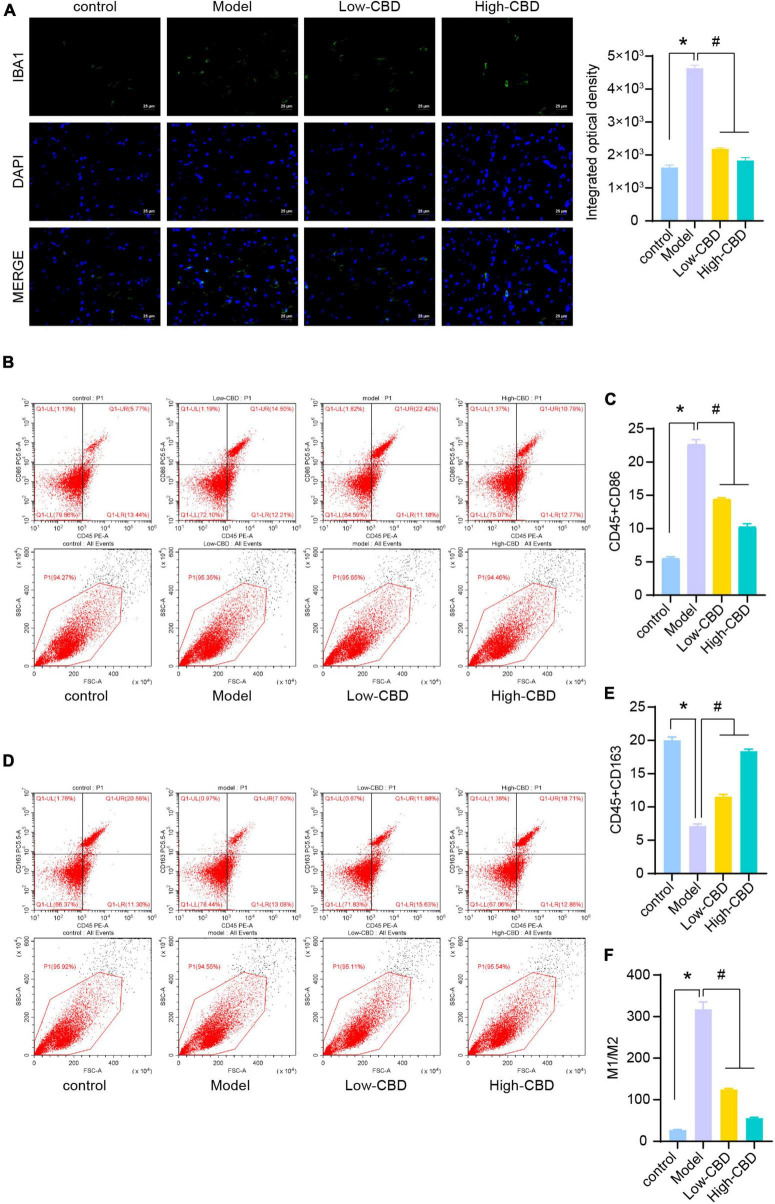
Effects of cannabidiol (CBD) on microglia polarization in epileptic rats. **(A)** IBA1 expression was detected by immunofluorescence (IF). **(B,C)** Analysis of M1 type microglia. CD45 + CD86 + double positive cells are expressed as M1 type microglia. **(D,E)** Analysis of M2 type microglia. CD45 + CD163 + double positive cells are expressed as M2 type microglia. **(F)** Statistical graph of M1/M2. *Represents significant comparison with the control group, and #represents significant comparison with the Model group, *P* < 0.05.

To explore the effect of CBD on epileptic rats, we applied the CBD treatment on epileptic rats. The IF results of IBA1 demonstrated that the low-and high-CBD groups were significantly lower than the model group (Figure 2A). FCM double staining was used to detect the expression of CD45 + CD86 (Figures 2B,C) and CD45 + CD163 (Figures 2D,E) to identify the content of M1 type and M2 type microglia cells, respectively. M1-type microglia cells were significantly decreased in the low-CBD and high-CBD groups compared to the Model group. However, the expression of M2-type cells was markedly increased. The ratio of M1/M2 was significantly decreased in the low-CBD and high-CBD groups compared with the Model group (Figure 2F).

### Cannabidiol reduces neuroinflammation in epileptic rats

**FIGURE 3:**
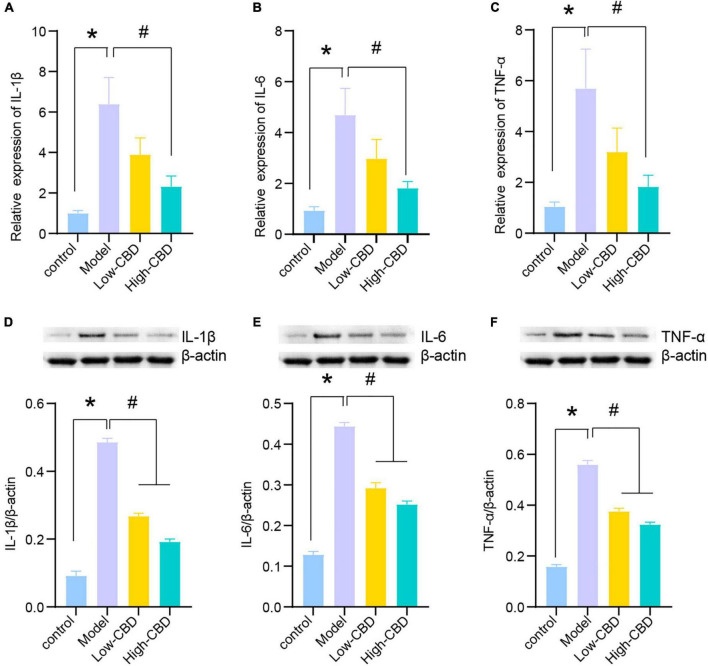
Effects of cannabidiol (CBD) on the expression of IL-1β, IL-6, and TNF-α in the temporal lobe cortex of epileptic rats. **(A-C)** IL-1β, IL-6, and TNF-α were detected by qRT-PCR. **(D-F)** IL-1β, IL-6, and TNF-α were detected by western blotting (WB). *Represents significant comparison with the control group, #represents significant comparison with the Model group, *P* < 0.05.

We subsequently determined the relative levels of proinflammatory factors in the brain tissues of epileptic rats. The expression levels of IL-1β, IL-6, and TNF-α in the model group were significantly higher than in the control group (Figures 3A–C). Thus, the levels of proinflammatory factors in the brains of epileptic rats were markedly increased. The low- and high-CBD groups were significantly lower than the model group, and the result from Western Blotting further demonstrated this (Figures 3D–F). The expression of IL-1β, IL-6, and TNF-α in the low-CBD and high-CBD groups was significantly lower than that in the model group. Moreover, the gene and protein expression of IL-1β, IL-6, and TNF-α were not significantly different in the CBZ group compared with the high-CBD group (Supplementary Figure 1).

**FIGURE 4:**
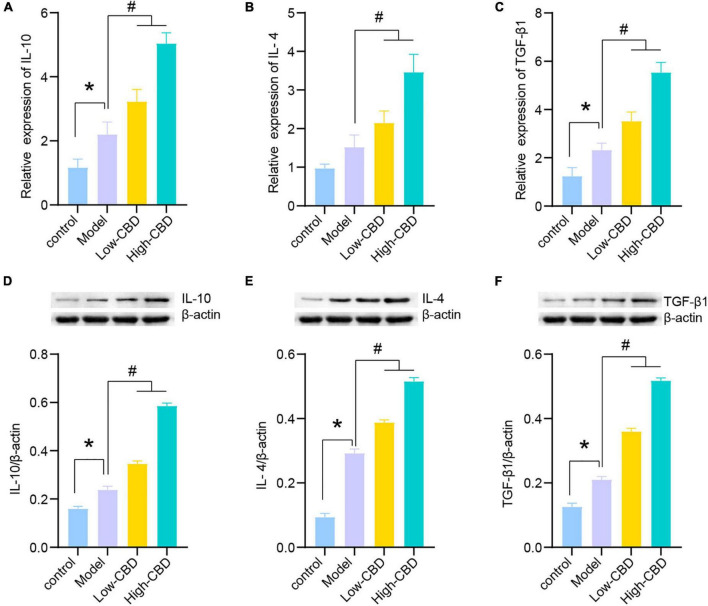
Effects of cannabidiol (CBD) on the expression of IL-10, IL-4, and TGF-β1 in the temporal lobe cortex of epileptic rats. **(A-C)** IL-10, IL-4, and TGF-β1 were detected by qRT-PCR. **(D-F)** IL-10, IL-4, and TGF-β1 were detected by western blotting (WB). *Represents significant comparison with the control group, #represents significant comparison with the Model group, *P* < 0.05.

The results of qRT-PCR (Figures 4A–C) and WB (Figures 4D–F) demonstrated the levels of anti-inflammatory factors, including IL-10, IL-4, and TGF-β1, in the brain of rats altered among the four groups. The CBD treatment group was significantly higher than the model group. The high-CBD group was higher than the low-CBD group. IL-10, IL-4, and TGF-β1 were lower in the CBZ than in the high-CBD groups (Supplementary Figure 1).

### Cannabidiol partially regulates gut microbiota in epileptic rats

**FIGURE 5:**
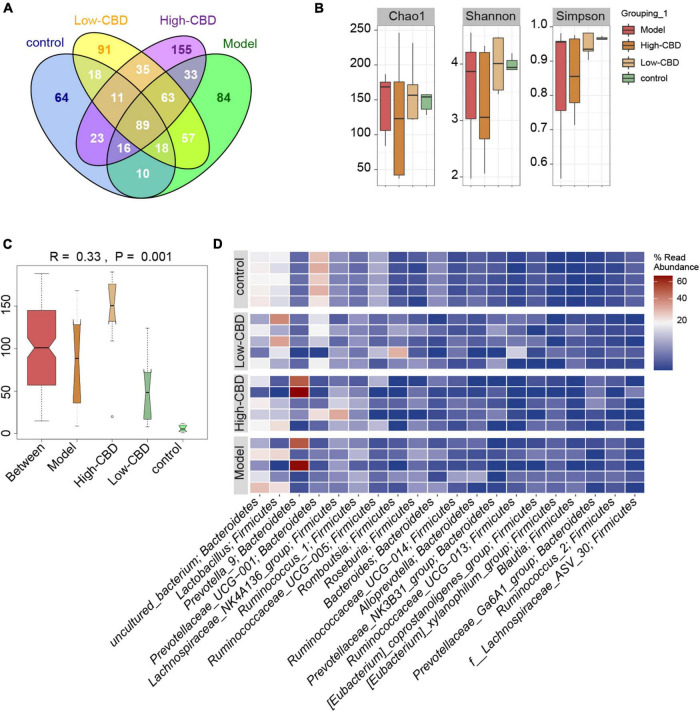
Effects of cannabidiol (CBD) on the gut microbiota structure in epileptic rats. **(A)** Venn diagram. **(B)** α-diversity analysis. **(C)** ANOSIM analysis. **(D)** The Heat Map visualization of the abundance and classification information of the top 20 ASVs. A color change from blue to red represents higher abundance.

16S rDNA sequencing was performed on rat fecal samples. A Venn diagram was used to visualize the four groups of common and unique amplicon sequence variants (ASVs) (Figure 5A). ASVs were used to identify species characteristics. 89 ASVs were common among the four groups. There were 64 unique ASVs in the control group, 84 in the model group, 91 in the low-CBD group, and 155 unique ASVs in the high-CBD group. There were no significant differences among all groups’ Chao1, Shannon, and Simpson indices. The results suggested no significant difference in the α-diversity of the gut microbiota of rats receiving different treatments (Figure 5B). However, ANOSIM analysis showed that the *R*-value was 0.33 and the *P*-value was 0.001 (Figure 5C), demonstrating that the intergroup difference exceeded the intragroup difference, and the grouping was meaningful. A Heat Map was used to visualize the classification information and relative abundance of the top 20 ASVs (Figure 5D).

**FIGURE 6:**
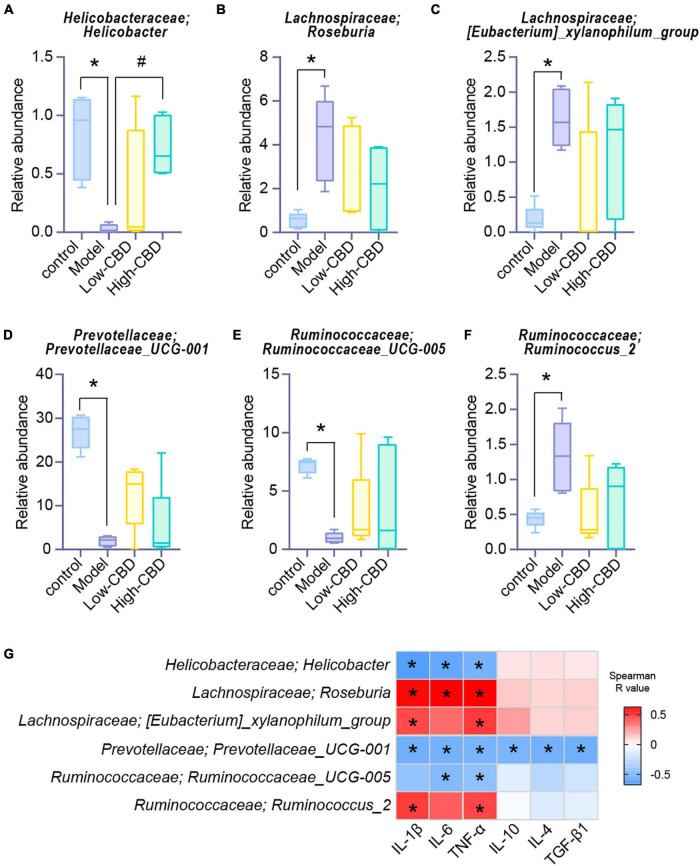
Effects of cannabidiol (CBD) on the changes of the abundance of gut microbiota in rats with Epilepsy. **(A-F)** At the genus level, the abundance of *Helicobacter*, *Roseburia*, *[Eubacterium]_xylanophilum_group*, *Prevotellaceae_UCG-001*, *Ruminococcaceae_UCG-005*, and *Ruminococcus_2*. *Represents significant comparison with the control group, #represents significant comparison with the Model group, *P* < 0.05. **(G)** Correlation analysis of gut bacteria and cytokines. Blue represents negative correlation, and red represents positive correlation; The larger the absolute value of R, the stronger the correlation; *indicates significant correlation.

We conducted a different analysis of gut microbiota at different levels to further clarify the influence of CBD on the abundance of specific microbiota in epileptic rats. At the genus level (Figures 6A–F), the abundance of *Helicobacter*, *Roseburia*, *Eubacterium_xylanophilum_group*, *Prevotellaceae_UCG-001*, *Ruminococcaceae_UCG-005*, and *Ruminococcus_2* were significantly different in the model group compared with the control group. However, the abundance of gut microbiota in epileptic rats was altered after the CBD intervention. Spearman’s rank correlation coefficient showed a significant correlation between the abundance of gut microbiota and inflammatory factors (Figure 6G). Furthermore, the abundances of *Prevotellaceae_UCG-001* were negatively correlated with proinflammatory and anti-inflammatory factors. The abundances of *Helicobacter* and *Ruminococcaceae_UCG-005* were negatively correlated with proinflammatory factors, and *Roseburia*, *Eubacterium_xylanophilum_group*, and *Ruminococcus_2* were positively correlated with proinflammatory factors. Consequently, we conclude that the structure of gut microbiota of epileptic rats is dysregulated, and CBD could promote gut microbiota remodeling or rebalance to some extent.

### Cannabidiol partially mediates gut microbiota metabolism in epileptic rats

**FIGURE 7:**
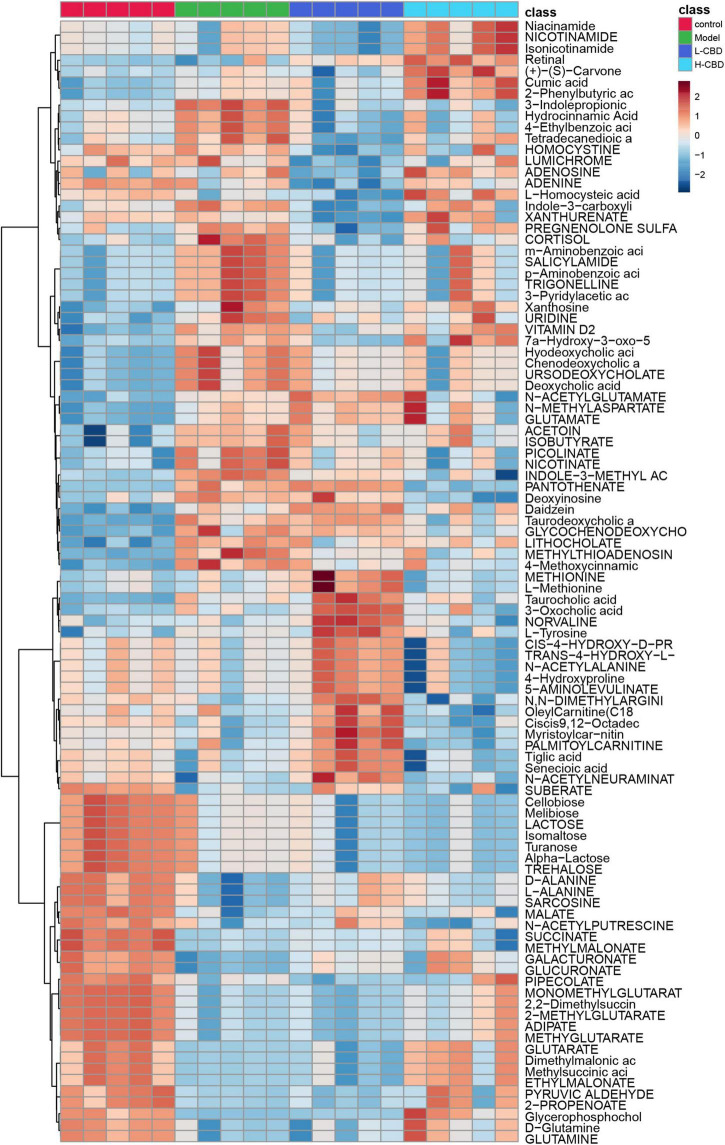
Differential metabolites abundance analysis. A change from blue to red indicates an increase in the levels of metabolite.

Finally, to explore whether CBD has an effect on the metabolism of rats with Epilepsy, we performed an untargeted metabolomics analysis of fecal samples from rats. A Heat Map was used to visualize the top 100 differential metabolites (Figure 7). It was clear that the abundance of metabolites differed among the four groups.

**FIGURE 8:**
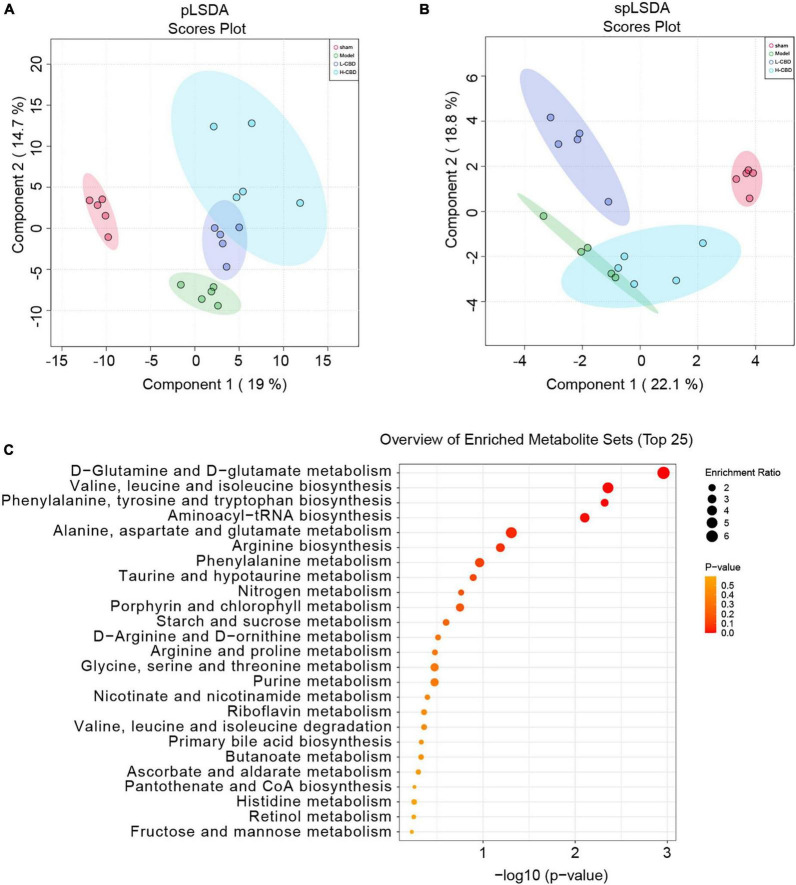
Effects of cannabidiol (CBD) on fecal metabolism in epileptic rats. **(A)** partial least squares discriminant analysis (pLSDA) analysis. **(B)** sparse partial least squares discriminant analysis (spLSDA) analysis. **(C)** Enrichment of the differential metabolic pathways. A color change from yellow to red represents the lower of the *P* value.

**FIGURE 9:**
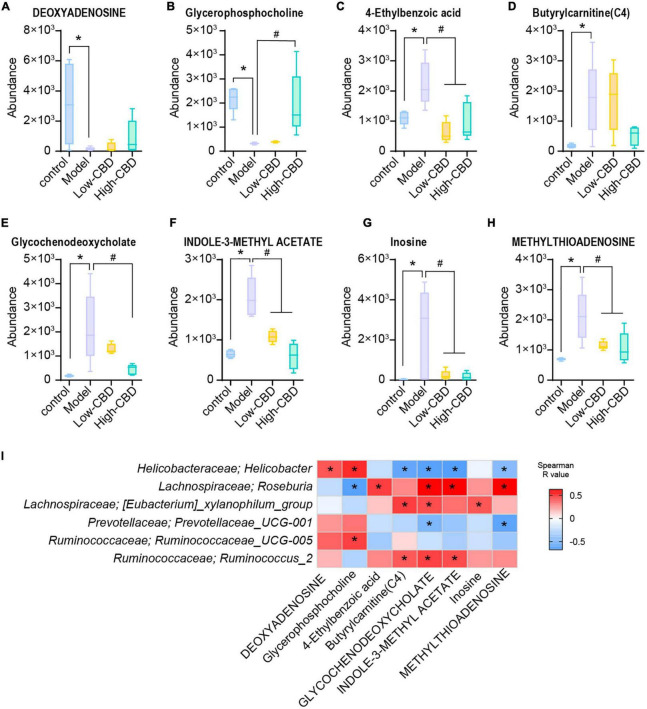
Effects of cannabidiol (CBD) on the changes of the levels of metabolites in rats with Epilepsy. **(A-H)** Relative abundance of deoxyadenosing, glycerophosphocholine, 4-ethylbenzoic acid, butyrylcarnitine (C4), glycochenodeoxycholate, indole-3-methyl acetate, inosine, and methylthioadenosine. *Indicates a significant difference compared with the control group and #indicates a significant difference compared with the model group, *P* < 0.05. **(I)** The relationship between the gut bacteria and the different metabolites. Blue represents negative correlation and red represents positive correlation. The larger the absolute value of R, the stronger the correlation; *indicates significant correlation.

The results of partial least squares discriminant analysis (pLSDA) (Figure 8A) and sparse partial least squares discriminant analysis (spLSDA) (Figure 8B) showed that the sample points of the control and model groups were far from each other. In the pLSDA, the sample points of the low-CBD and high-CBD groups were far from those of the model group. In the spLSDA, the low-CBD and high-CBD sample points were far apart. The results demonstrated that the four groups of samples were separated. A bubble diagram was used to visualize the different metabolic pathways enriched in the top 25 (Figure 8C). Among these, the functional pathways of metabolism and genetic information processing at the L1 level were altered. The D-glutamine and D-glutamate metabolism; valine, leucine, and isoleucine biosynthesis; aminoacyl-tRNA biosynthesis; arginine biosynthesis; phenylalanine, tyrosine, and tryptophan biosynthesis; and phenylalanine metabolism were significantly different at the L3 level. We then analyzed the levels of metabolites (Figures 9A–H). Glycerophosphocholine (Figure 9B) was lower in the model and low-CBD groups compared with the control group but increased significantly in the high-CBD group. The levels of several metabolites, including 4-ethylbenzoic acid (Figure 9C), glycochenodeoxycholate (Figure 9E), indole-3-methyl acetate (Figure 9F), inosine (Figure 9G) and methylthioadenosine (Figure 9H) were significantly increased in the model group compared with those in the control group, while the levels of metabolites in the CBD treatment group were significantly lower compared to those in the model group.

The abundance of *Helicobacter*, *Roseburia*, *[Eubacterium]_xylanophilum_group*, and *Ruminococcus_2* was significantly associated with multiple metabolites at the genus level (Figure 9I). Among them, the levels of the metabolites glycerophosphocholine, butyrylcarnitine (C4), glycochenodeoxycholate, indole-3-methyl acetate, and methylthioadenosine were correlated with different bacterial communities.

## Discussion

Cannabidiol (CBD), a major non-psychoactive compound derived from cannabis, can potentially treat neuropsychiatric diseases (26). In this study, CBD treatment alleviated neuroinflammation in brain tissues of epileptic rats, reduced M1/M2 and played a neuroprotective role. In addition, we found that CBD may play a role in treating epileptic rats by regulating the gut microbiota and related metabolism.

The role of CBD in alleviating Epilepsy is well-known (27). Mori et al. found that CBD plays a neuroprotective role by reducing glial cell responses (28). This finding is consistent with the results of our study, where the number of microglial cells and proinflammatory M1 type positive cells decreased, and the number of anti-inflammatory M2 type positive cells increased in epileptic rats treated with CBD. This suggests that CBD promotes microglial polarization into the M2 type. Furthermore, the expression of proinflammatory factors, including IL-6 and TNF-α, can be reduced to alleviate neuronal injury and play a neuroprotective role (29). We also found that the levels of IL-1β, IL-6, and TNF-α decreased, and levels of anti-inflammatory cytokines IL-10, TGF-β1, and IL-4 [regulatory factor with adaptive immunity (30)] increased. These confirmed the effectiveness of CBD in anti-neuroinflammation in rats with Epilepsy.

The gut microbiota plays an important role in maintaining the stability of the gut barrier and resisting pathogen invasion (31). Several studies have found that gut microbiota plays an important role in the pathophysiology of nervous system diseases, including spinal cord injury (SCI) (32), neuromyelitis Optica (NMO) (33), Alzheimer’s disease (AD) (34), and multiple sclerosis (MS) (35). şafak et al. demonstrated that dysregulation of gut microbiota could affect the development of Epilepsy (36). In our study, the abundance of *Helicobacter*, *Prevotellaceae_UCG-001*, and *Ruminococcaceae_UCG-005* was significantly decreased in epileptic rats, whereas the abundance of *Roseburia*, *[Eubacterium]_xylanophilum_group*, and *Ruminococcus_2* was significantly increased. Exposure to various compounds, including drugs for Epilepsy, can positively or negatively alter the gut microbiota and reduce or exacerbate seizures (37). Although some antiepileptic drugs affect the growth and metabolism of gut bacteria, for example, lamotrigine suppresses ribosome biogenesis in *E. coli*, and thus may restrain its growth (38), there is little evidence of direct interaction between antiepileptic drugs and the gut microbiome. In our study, we found that the composition of the gut microbiota of epileptic rats was disordered. CBD treatment was beneficial for the restoration of at least part of the gut bacterial abundance in epileptic rats, such as *Prevotellaceae_UCG-001*. To the best of our knowledge, *Prevotellaceae UCG-001* is an SCFA-producing bacterium that plays an anti-inflammatory role in immune cells and inhibits the growth of invasive pathogens (39). This is an encouraging finding, suggesting that CBD may improve Epilepsy through beneficial gut bacteria.

Moreover, study has found that the gut microbiome can affect the occurrence and development of epilepsy by regulating the polarization of microglia (40). Activation of the M2 phenotype could suppress subsequent inflammation in epilepsy (40). Combined with the above analysis, microglia M2 polarization might be involved in the process of gut microbiome affecting epileptic seizures. Recent study has found that modulating the microbiome can affect the expression of IL-1β, IL-6, and TNF-α (41). Transplantation of a disturbed gut microbiome exacerbates inflammatory damage, including stimulating the expression of pro-inflammatory factors IL-1β, IL-6, and TNF-α, and reducing the levels of anti-inflammatory IL-10, IL-4, and TGF-β (42). From this, we could speculate that the gut microbiome might affect epileptic seizures by regulating the expression of inflammatory factors while regulating M2 polarization.

Gut microbiota produces a variety of substances that alter the excitation-inhibitory balance of the nervous system, including metabolites that act as neuromodulators (37). Correlation analysis showed that glycochenodeoxycholate levels were significantly correlated with different gut microbiota. Glycochenodeoxycholate is an important component of bile acids and is involved in steatosis and poor glucose tolerance (43). Gut microbiota can produce bioactive metabolites that directly or indirectly affect host physiology and metabolic balance (44). Dysfunctional glutamate metabolism in astrocytes can directly lead to neuronal over-excitation, which plays an important role in the pathogenesis of Epilepsy (45). Administration of valine, leucine, isoleucine, and branched-chain amino acids to epileptic rats increases the average latency period of epileptic seizures (46). In addition, mitochondrial aminoacyl-tRNA synthetases (AaRSs) provide amino acids homologous to tRNAs and regulate a variety of cellular processes. One study showed that mutations in human mitochondrial AaRSs cause epilepsy in infants (47). Acute supplementation of branched-chain amino acids (valine, leucine, and isoleucine) reduced seizures, whereas long-term oral supplementation with branched-chain amino acids led to worsening seizures (48). We also found a statistical difference in different groups’ enrichment of D-glutamine and D-glutamate metabolism, valine, leucine, isoleucine biosynthesis, and aminoacyl-tRNA biosynthesis. Hence, we hypothesized that CBD might be involved in regulating the metabolic function of the gut microbiota of epileptic rats. Furthermore, the composition of the gut microbiome and the levels of IL-10, IL-4, and TNF-α were altered in mice after drug intervention, and regulation of SCFAs, microbial metabolites, could affect the expression of IL-6 (49). Combined with the above analysis, we could speculate that the gut microbiome is involved in the process of CBD alleviating epilepsy, which might be mediated by microbial metabolites regulating inflammatory factors.

There were some limitations to our study. Although there are some network and neurochemical similarities between human TLE and Li-pilocarpine models, including increased neurotrophins and cognitive and memory disorders (50, 51), because these models have not been fully validated clinically, they cannot predict clinical responses to all treatment strategies. In addition, owing to the inherent limitations of the Li-pilocarpine model, the frequency and severity of induced spontaneous seizures vary, which may lead to unavoidable systematic errors. Therefore, in the selection of samples and sample size, the CBD group alone could be set, relevant clinical samples could be supplemented, and the number of samples could be increased. Furthermore, animal behavioral tests, untargeted metabolic analysis of blood, and targeted analysis of related metabolites could be supplemented to obtain more comprehensive data. In the future, we intend to combine clinical samples and fecal microbiota transplantation (FMT) to further study the mechanism of CBD and gut microbiota in Epilepsy.

## Conclusion

In conclusion, CBD could effectively inhibit neuroinflammation in epileptic rats. Furthermore, CBD might have a tendency to promote gut microbiota remodeling and altered the metabolic pathways in the gut of epileptic rats. It is not yet clear whether CBD manipulates the gut microbiota to improve the symptoms of Epilepsy. We hypothesized that the improvement of CBD in Epilepsy might be through changes induced in the gut microbiota and will continue to explore this area in further research.

## Data availability statement

The datasets presented in this study can be found in online repositories. The names of the repository/repositories and accession number(s) can be found below: https://www.ncbi.nlm.nih.gov/, PRJNA735800.

## Ethics statement

This animal study was reviewed and approved by the Animal Ethical and Welfare Committee and The Second Xiangya Hospital, Central South University (No. 2021523).

## Author contributions

LqL and DM conceived and designed the experiments. LjL and XL analyzed the data. JXu and JXi prepared the figures. XG performed the experiments and drafted the work or revised it critically for important content. All authors contributed to manuscript revision, read, and approved the submitted version.

## Footnotes

1
http://www.bioinformatics.com.cn/static/others/jvenn/example.html

2
https://www.metaboanalyst.ca/

## Supplementary material

Supplementary Figure 1 After cannabidiol (CBD) and carbamazepine (CBZ) treatment, gene and protein expression of IL-1β, IL-6, TNF-α, IL-10, IL-4, and TGF-β1 was determined. *Represents significant comparison with the control group, #represents significant comparison with the Model group, and represents significant comparison with the high-CBD group, *P* < 0.05.
