# Supplementary figures and images for: Neuroprotection of cannabidiol in epileptic rats: Gut microbiome and metabolome sequencing
Source: Front Nutr. 2022 Nov 16;9:1028459. doi: 10.3389/fnut.2022.1028459 (PMC9709218; doi:10.3389/fnut.2022.1028459)

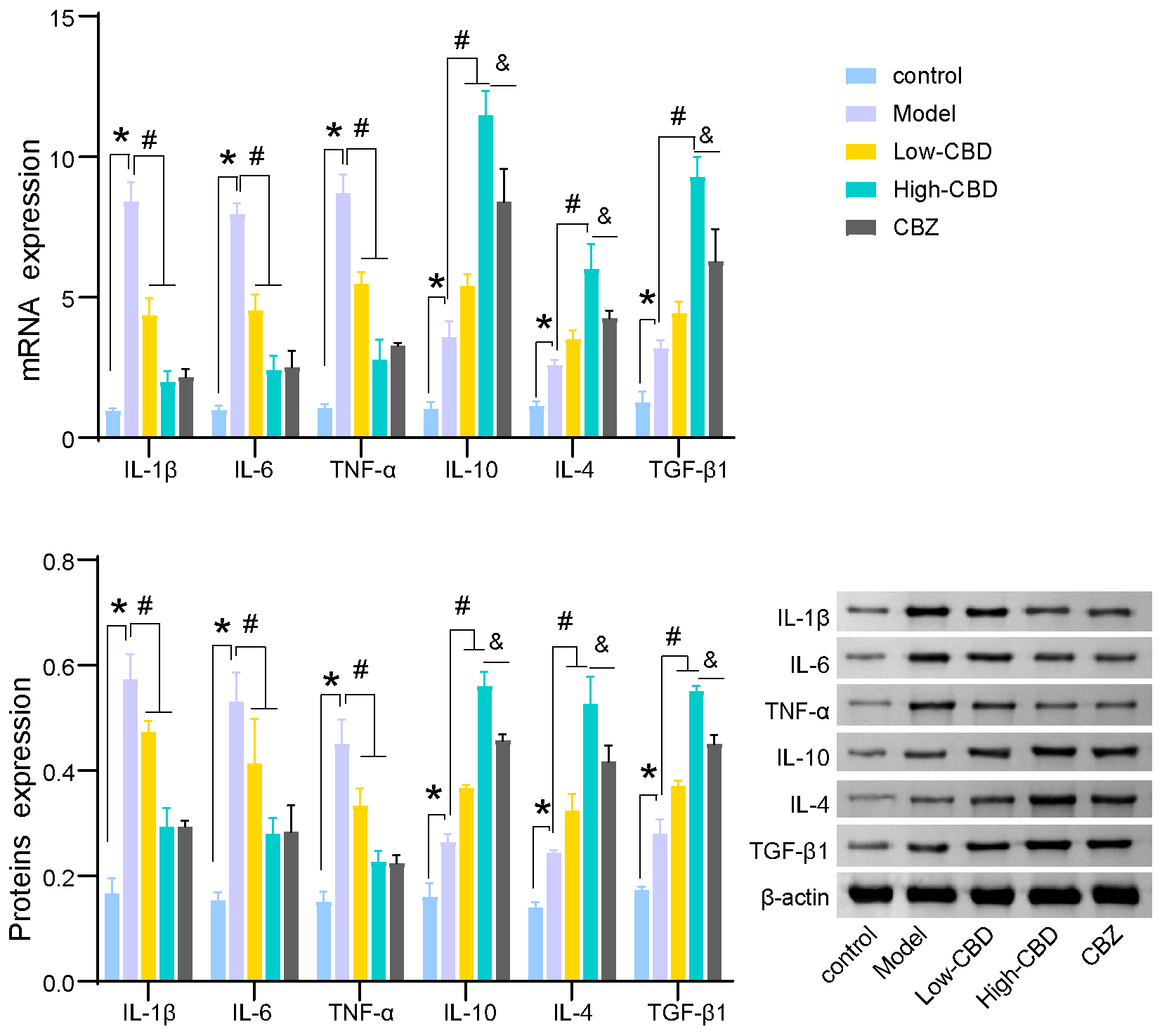

Supplement: Supplementary Figure 1 — After cannabidiol (CBD) and carbamazepine (CBZ) treatment, gene and protein expression of IL-1β, IL-6, TNF-α, IL-10, IL-4, and TGF-β1 was determined. *Represents significant comparison with the control group, #represents significant comparison with the Model group, and represents significant comparison with the high-CBD group, P < 0.05. [file Image_1.tif]
